# Crystal structure of aqua­(2-{[2-({2-[bis­(carboxyl­ato-κ*O*-meth­yl)amino-κ*N*]eth­yl}(carboxyl­ato-κ*O*-meth­yl)amino-κ*N*)eth­yl](carb­oxy­meth­yl)aza­niumyl}acetato)­gallium(III) trihydrate

**DOI:** 10.1107/S2056989018009428

**Published:** 2018-07-06

**Authors:** Martin Wallin, Peter Turner, Andrew Katsifis, Mingshi Yang, Hak-Kim Chan

**Affiliations:** a Advanced Drug Delivery Group, School of Pharmacy, University of Sydney, NSW, 2006, Australia; b School of Chemistry, University of Sydney, NSW 2006, Australia; c Department of PET & Nuclear Medicine, Royal Prince Alfred Hospital, NSW 2050, Australia; d Department of Pharmacy, University of Copenhagen, Universitetsparken 2, DK-2100, Copenhagen, Denmark

**Keywords:** crystal structure, gallium radioisotopes, chelating agents, pentetic acid, DTPA

## Abstract

In the title Ga^III^ complex compound with pentetic acid, [Ga(C_14_H_20_N_3_O_10_)(H_2_O)]·3H_2_O, the Ga^III^ centre is bound in a slightly distorted octa­hedral coordination sphere by two amine N atoms, three carboxyl­ate O atoms and one water O atom. The complex mol­ecule exists as a zwitterion. In the crystal, the complexes are linked to each other *via* O—H⋯O and C—H⋯O hydrogen bonds, forming layers parallel to (001). Three uncoordinating water mol­ecules link the complex layers *via* O—H⋯O, N—H⋯O and C—H⋯O hydrogen bonds, forming a three-dimensional network.

## Abstract 2

The structure of a Ga^III^ complex compound with pentetic acid is reported. The complex mol­ecule is a zwitterion and the Ga^III^ centre is bound in a slightly distorted octa­hedral coordination sphere by two amine N atoms, three carboxyl­ate O atoms and one water O atom.

## Chemical context   

**Figure 1:**
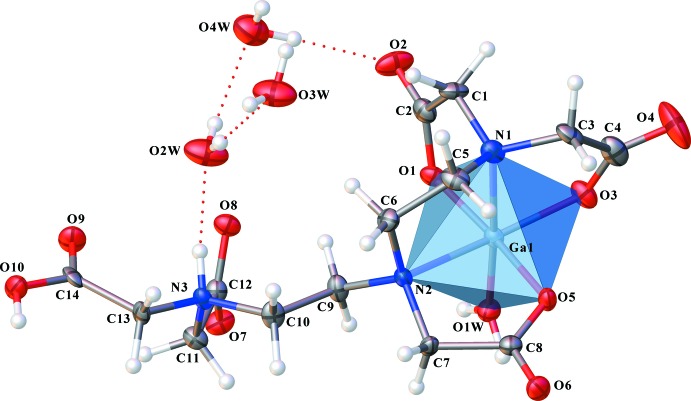
OLEX*2* generated depiction of the title compound, with displacement ellipsoids drawn at the 75% probability level. Dashed lines show O—H⋯O and N—H⋯O hydrogen bonds.

The use of gallium-68 (^68^Ga) for mol­ecular imaging of diseases has become increasingly popular and the number of ^68^Ga-related articles has increased drastically in the past 10 years, as pointed out by Velikyan (2014 ▸). The application span is wide and covers the diagnosis of cancer, cardiovascular disease, infection and inflammatory conditions (Brasse & Nonat, 2015 ▸; Jalilian & Akhlaghi, 2013 ▸; Banerjee & Pomper, 2013 ▸; Schultz *et al.*, 2013 ▸). The increase in popularity and use can be ascribed to several factors. On the one hand, ^68^Ga produces high-quality PET images. On the other hand, it has a half-life of 68 min, which makes it suitable for use in patients as the radiation dose can be kept at a minimum (Hofman & Hicks, 2016 ▸). ^68^Ga can be eluted from a ^68^Ge/^68^Ga generator multiple times a day, which makes it easy for hospitals to prepare gallium solutions for patients on demand. It is vital that gallium ions are complexed, as free ions may cause undesirable effects *in vivo*. First, free gallium can cause iron release from transferrin, which may cause free-radical toxicity. Second, gallium ions may cause an additional and unnecessary radiation dose. 2-(Bis{2-[bis­(carb­oxy­meth­yl)amino]­eth­yl}amino)­acetic acid (pentetic acid or DTPA) is an amino-polycarb­oxy­lic acid consisting of a di­ethyl­enetri­amine backbone with five carb­oxy groups. A complex is easily formed between gallium and DTPA and it has a stability constant of 10^23.32^, which makes the complex stable against exchange with transferrin (Moerlein & Welch, 1981 ▸; Green & Welch, 1989 ▸). DTPA-peptides labelled with ^68^Ga have been used for liver-function imaging, determination of low-density lipoprotein metabolism, bone-marrow function and mol­ecular identification of metastatic tumours (Haubner *et al.*, 2013 ▸; Moerlein *et al.*, 1991 ▸; Vera *et al.*, 2012 ▸; Pitalúa-Cortés *et al.*, 2017 ▸), but the mol­ecular structure of our compound has not yet been reported. Here we present and describe the mol­ecular structure of the title compound (Fig. 1 ▸).
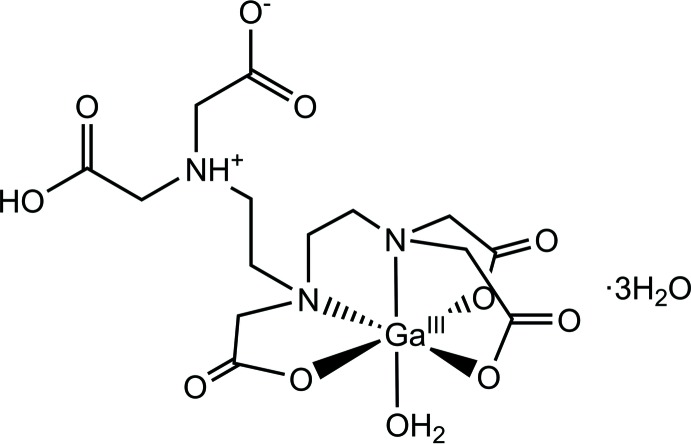


## Structural commentary   

The complex mol­ecule (abbreviated as Ga-DTPA) is a zwitterion and has a slightly distorted octa­hedral coordination geometry with one water and one amine in the axial positions, and three carboxyl­ate groups and one amine in the equatorial positions. The complex consists of three five-membered Ga/N/C/C/O chelate rings and one five-membered Ga/N/C/C/N chelate ring. The Ga—N bonds [Ga1—N1 = 2.081 (4) Å and Ga1—N2 = 2.156 (3) Å] are significantly longer than the Ga—O bonds [Ga1—O1 = 1.933 (3) Å, Ga1—O3 = 1.925 (3) Å, Ga1—O5 = 1.964 (3) Å and Ga1—O1*W* = 1.916 (3) Å]. The C—O bond lengths coordinating to the Ga^III^ atom vary little, with the shortest and longest bonds differing by only 0.019 Å [C2—O1 = 1.286 (5) Å, C4—O3 = 1.305 (5) Å and C8—O5 = 1.293 (5) Å]. The three *trans* angles, N1—Ga1—O1*W*, O1—Ga1—O5 and O3—Ga1—N2, are 174.57 (16), 174.05 (12) and 164.97 (13)°, respectively. The O—Ga—O, O—Ga—N and N—Ga—N bite angles in the chelate rings deviate somewhat from 90°, ranging from 81.75 (12) to 95.91 (12)°.

## Supra­molecular features   

**Figure 2:**
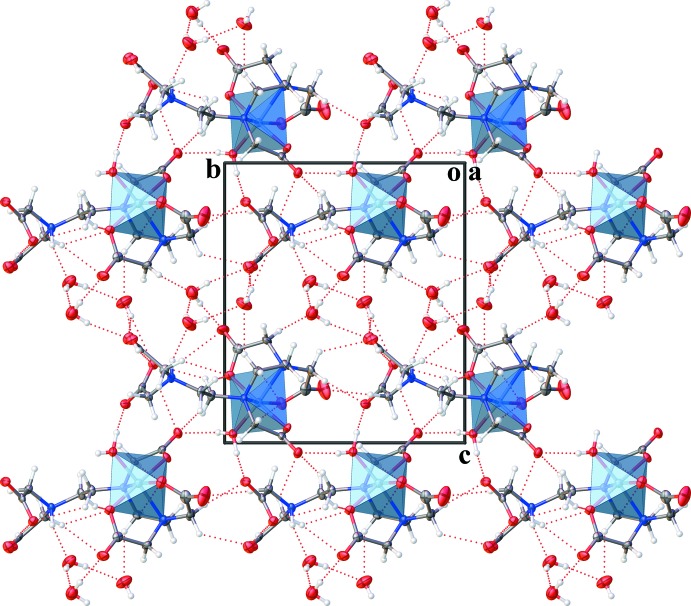
A packing diagram of the title compound, viewed along the *a* axis. Dashed lines show O—H⋯O, N—H⋯O and C—H⋯O hydrogen bonds.

Packing depictions viewed along the *a* and *b* axes provided in Figs. 2 ▸ and 3 ▸, respectively, show pairs of layers containing the complexes parallel to the (001) plane. In the layer, the complexes are linked to each other by O—H⋯O and C—H⋯O hydrogen bonds (Table 1 ▸). Three uncoordinating water mol­ecules link the complex layers *via* O—H⋯O, N—H⋯O and C—H⋯O hydrogen bonds, forming a three-dimensional network.

## Database survey   

In our survey of the Cambridge Structural Database (CSD version 5.39, update November 2017; Groom *et al.*, 2016 ▸), we found 64 crystal structures of metal complexes with DTPA. In another search, we found 72 crystal structures of gallium complexes hexa-coordinated by two N and four O atoms.

## Synthesis and crystallization   

**Figure 4:**
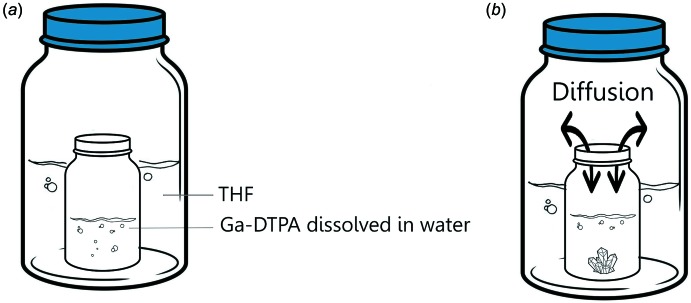
Vapor liquid diffusion technique illustration. (*a*) a HPLC vial containing Ga-DTPA dissolved in water was placed inside a bigger vial. The closed bigger vial contained THF. (*b*) THF diffused slowly into the small vial. After four weeks, visible Ga-DTPA crystals were formed.

DTPA (50 mg) in acetate buffer (2 mL) adjusted to pH = 4.2 was heated with stirring for dissolution. Gallium nitrate (39.9 mg) was then added to the DTPA solution and the mixture was stirred for at least 10 min at 353 K. The solution was concentrated under ambient pressure at room temperature. When almost all of the solvent had evaporated, methanol was added dropwise to precipitate Ga-DTPA. The precipitate was collected on a 0.22 µm polyamide filter and dried at room temperature. The obtained Ga-DTPA (1.30 mg) was re-dissolved in ultra-pure water (1 mL) and single crystals suitable for X-ray diffraction were obtained after four weeks by slow diffusion of tetra­hydro­furan into the aqueous solution, as illustrated in Fig. 4 ▸.

## Refinement   

Crystal data, data collection and structure refinement details are summarized in Table 2 ▸. N- and O-bound H atoms were located in difference-Fourier maps and freely refined. C-bound H atoms were positioned geometrically (C—H = 0.99 Å) and refined using a riding model with *U*_iso_(H) = 1.2*U*_eq_(C).

## Supplementary Material

Crystal structure: contains datablock(s) I. DOI: 10.1107/S2056989018009428/is5497sup1.cif

Structure factors: contains datablock(s) I. DOI: 10.1107/S2056989018009428/is5497Isup2.hkl

CCDC reference: 1852608

Additional supporting information:  crystallographic information; 3D view; checkCIF report

## Figures and Tables

**Figure 3:**
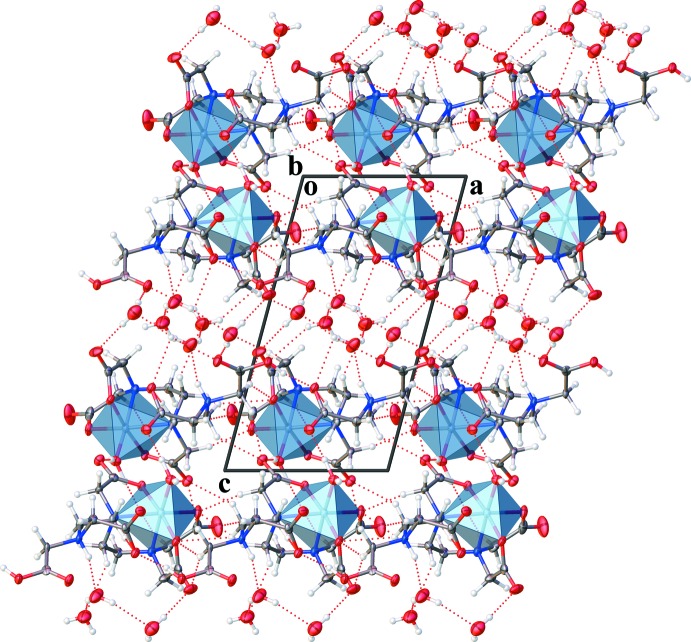
A packing diagram of the title compound, viewed along the *b* axis. Dashed lines show O—H⋯O, N—H⋯O and C—H⋯O hydrogen bonds.

**Table 1:** Hydrogen-bond geometry (Å, °)

*D*—H⋯*A*	*D*—H	H⋯*A*	*D*⋯*A*	*D*—H⋯*A*
O10—H10*O*⋯O8^i^	0.87 (6)	1.67 (6)	2.525 (4)	169 (6)
O1*W*—H1*WA*⋯O6^ii^	0.71 (5)	1.93 (5)	2.636 (5)	174 (6)
O1*W*—H1*WB*⋯O7^iii^	0.99 (6)	1.54 (6)	2.524 (5)	174 (5)
O2*W*—H2*WA*⋯O3*W*	0.84 (7)	1.94 (7)	2.741 (5)	158 (6)
O2*W*—H2*WB*⋯O4*W*	0.77 (6)	2.09 (6)	2.828 (5)	160 (6)
O3*W*—H3*WA*⋯O2^i^	0.79 (10)	2.47 (10)	2.934 (5)	119 (9)
O3*W*—H3*WA*⋯O10^iv^	0.79 (10)	2.37 (10)	3.096 (5)	153 (9)
O3*W*—H3*WB*⋯O8^v^	0.81 (7)	2.60 (6)	3.215 (5)	134 (5)
O3*W*—H3*WB*⋯O9^v^	0.81 (7)	2.28 (6)	2.934 (5)	138 (6)
O4*W*—H4*WA*⋯O2	0.80 (6)	2.00 (6)	2.806 (5)	175 (6)
O4*W*—H4*WB*⋯O9^v^	0.83 (7)	2.09 (7)	2.911 (5)	168 (7)
N3—H3*N*⋯O2*W*	0.90 (5)	1.91 (5)	2.737 (5)	152 (4)
C1—H1*A*⋯O4*W* ^vi^	0.99	2.43	3.417 (6)	173
C3—H3*A*⋯O7^vii^	0.99	2.25	3.197 (5)	159
C3—H3*B*⋯O10^viii^	0.99	2.52	3.225 (5)	128
C6—H6*B*⋯O3*W*	0.99	2.53	3.254 (5)	130
C7—H7*B*⋯O3^i^	0.99	2.28	3.227 (5)	161
C9—H9*B*⋯O8	0.99	2.53	3.207 (5)	126
C10—H10*A*⋯O6^ix^	0.99	2.46	3.271 (5)	139
C10—H10*B*⋯O1^i^	0.99	2.53	3.300 (5)	134
C11—H11*A*⋯O4^x^	0.99	2.45	3.438 (5)	176
C13—H13*A*⋯O1^i^	0.99	2.54	3.367 (5)	140
C13—H13*A*⋯O2^i^	0.99	2.41	3.368 (5)	162
C13—H13*B*⋯O6^ix^	0.99	2.34	3.150 (5)	139

Symmetry codes: (i) 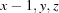; (ii) 
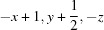
; (iii) 
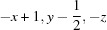
; (iv) 
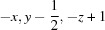
; (v) 
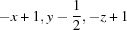
; (vi) 
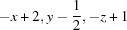
; (vii) 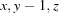; (viii) 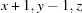; (ix) 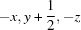; (x) 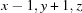.

**Table 2:** Experimental details

Crystal data	
Chemical formula	[Ga(C_14_H_20_N_3_O_10_)(H_2_O)]·3H_2_O
*M*_r_	532.11
Crystal system, space group	Monoclinic, *P*2_1_
Temperature (K)	100
*a*, *b*, *c* (Å)	7.1477 (2), 11.0616 (3), 13.3460 (4)
β (°)	104.929 (3)
*V* (Å^3^)	1019.58 (5)
*Z*	2
Radiation type	Mo *K*α
μ (mm^−1^)	1.43
Crystal size (mm)	0.13 × 0.06 × 0.03

Data collection	
Diffractometer	Oxford Diffraction SuperNova Dual Source diffractometer with an Atlas detector
Absorption correction	Multi-scan (*CrysAlis PRO*; Rigaku OD, 2015 ▸)
*T*_min_, *T*_max_	0.915, 1.00
No. of measured, independent and observed [*I* > 2σ(*I*)] reflections	23585, 6213, 5124
*R*_int_	0.073
(sin θ/λ)_max_ (Å^−1^)	0.714

Refinement	
*R*[*F* ^2^ > 2σ(*F* ^2^)], *wR*(*F* ^2^), *S*	0.044, 0.073, 1.05
No. of reflections	6213
No. of parameters	329
No. of restraints	1
H-atom treatment	H atoms treated by a mixture of independent and constrained refinement
Δρ_max_, Δρ_min_ (e Å^−3^)	0.73, −0.44
Absolute structure	Flack *x* determined using 2002 quotients [(*I* ^+^)−(*I* ^−^)]/[(*I* ^+^)+(*I* ^−^)] (Parsons *et al.*, 2013 ▸)
Absolute structure parameter	−0.009 (7)

Computer programs: *CrysAlis PRO* (Rigaku OOD, 2015 ▸), *SHELXT* (Sheldrick, 2015*a* ▸), *SHELXL2018* (Sheldrick, 2015*b* ▸), *OLEX2* (Dolomanov *et al.*, 2009 ▸), *WinGX* (Farrugia, 2012 ▸).

## supplementary crystallographic information

### Crystal data

**Table:**

[Ga(C_14_H_20_N_3_O_10_)(H_2_O)]·3H_2_O	*F*(000) = 552
*M**_r_* = 532.11	*D*_x_ = 1.733 Mg m^−^^3^
Monoclinic, *P*2_1_	Mo *K*α radiation, λ = 0.71073 Å
Hall symbol: P 2yb	Cell parameters from 5696 reflections
*a* = 7.1477 (2) Å	θ = 3.7–28.0°
*b* = 11.0616 (3) Å	µ = 1.43 mm^−^^1^
*c* = 13.3460 (4) Å	*T* = 100 K
β = 104.929 (3)°	Blade, colourless
*V* = 1019.58 (5) Å^3^	0.13 × 0.06 × 0.03 mm
*Z* = 2	

### Data collection

**Table:**

Oxford Diffraction SuperNova Dual Source diffractometer with an Atlas detector	6213 independent reflections
Radiation source: micro-focus sealed X-ray tube	5124 reflections with *I* > 2σ(*I*)
Mirror monochromator	*R*_int_ = 0.073
Detector resolution: 10.5861 pixels mm^-1^	θ_max_ = 30.5°, θ_min_ = 3.2°
ω scans	*h* = −10→10
Absorption correction: multi-scan (*CrysAlis PRO*; Rigaku OD, 2015)	*k* = −15→15
*T*_min_ = 0.915, *T*_max_ = 1.00	*l* = −19→19
23585 measured reflections	

### Refinement

**Table:**

Refinement on *F*^2^	Hydrogen site location: mixed
Least-squares matrix: full	H atoms treated by a mixture of independent and constrained refinement
*R*[*F*^2^ > 2σ(*F*^2^)] = 0.044	*w* = 1/[σ^2^(*F*_o_^2^) + (0.02*P*)^2^] where *P* = (*F*_o_^2^ + 2*F*_c_^2^)/3
*wR*(*F*^2^) = 0.073	(Δ/σ)_max_ < 0.001
*S* = 1.05	Δρ_max_ = 0.73 e Å^−^^3^
6213 reflections	Δρ_min_ = −0.44 e Å^−^^3^
329 parameters	Absolute structure: Flack *x* determined using 2002 quotients [(*I*^+^)-(*I*^-^)]/[(*I*^+^)+(*I*^-^)] (Parsons *et al.*, 2013)
1 restraint	Absolute structure parameter: −0.009 (7)

### Special details

**Table:**

Geometry. All esds (except the esd in the dihedral angle between two l.s. planes) are estimated using the full covariance matrix. The cell esds are taken into account individually in the estimation of esds in distances, angles and torsion angles; correlations between esds in cell parameters are only used when they are defined by crystal symmetry. An approximate (isotropic) treatment of cell esds is used for estimating esds involving l.s. planes.

### Fractional atomic coordinates and isotropic or equivalent isotropic displacement parameters (Å^2^)

**Table:**

	*x*	*y*	*z*	*U*_iso_*/*U*_eq_	
Ga1	0.67818 (6)	0.35920 (4)	0.14406 (3)	0.00910 (10)	
C1	0.8057 (6)	0.3244 (4)	0.3657 (3)	0.0126 (9)	
H1A	0.919556	0.274628	0.399527	0.015*	
H1B	0.729278	0.337981	0.416943	0.015*	
O1	0.8169 (4)	0.4789 (3)	0.2405 (2)	0.0112 (6)	
O2	0.9839 (4)	0.5070 (3)	0.4037 (2)	0.0192 (7)	
C2	0.8752 (6)	0.4454 (4)	0.3358 (3)	0.0119 (9)	
O3	0.9021 (4)	0.2641 (3)	0.1407 (2)	0.0143 (7)	
C3	0.7772 (6)	0.1419 (4)	0.2575 (3)	0.0143 (9)	
H3A	0.676921	0.084101	0.220995	0.017*	
H3B	0.844674	0.105973	0.325046	0.017*	
O4	1.0435 (5)	0.0862 (3)	0.1905 (3)	0.0312 (9)	
C4	0.9226 (6)	0.1631 (4)	0.1929 (4)	0.0164 (10)	
O5	0.5143 (4)	0.2406 (3)	0.0522 (2)	0.0101 (6)	
C5	0.4793 (6)	0.2422 (4)	0.2793 (3)	0.0120 (9)	
H5A	0.474766	0.213062	0.348815	0.014*	
H5B	0.413340	0.181905	0.227326	0.014*	
O6	0.2129 (4)	0.1952 (3)	−0.0352 (2)	0.0132 (6)	
C6	0.3768 (5)	0.3630 (5)	0.2572 (3)	0.0113 (7)	
H6A	0.237081	0.351202	0.251339	0.014*	
H6B	0.428383	0.418161	0.316320	0.014*	
O7	0.5490 (4)	0.9137 (3)	0.1386 (2)	0.0133 (6)	
C7	0.2595 (5)	0.3761 (4)	0.0650 (3)	0.0086 (9)	
H7A	0.237373	0.439360	0.010694	0.010*	
H7B	0.134525	0.359646	0.081351	0.010*	
O8	0.5857 (4)	0.7981 (3)	0.2800 (2)	0.0120 (6)	
C8	0.3304 (6)	0.2617 (4)	0.0239 (3)	0.0098 (8)	
O9	0.2242 (4)	0.9005 (3)	0.3733 (2)	0.0154 (7)	
O10	−0.0876 (4)	0.8862 (3)	0.3686 (2)	0.0134 (7)	
H10O	−0.199 (8)	0.861 (7)	0.331 (4)	0.054 (18)*	
O1W	0.6862 (5)	0.4657 (3)	0.0320 (3)	0.0124 (7)	
C14	0.0674 (5)	0.8541 (5)	0.3389 (3)	0.0106 (7)	
C13	0.0341 (6)	0.7512 (4)	0.2622 (3)	0.0100 (8)	
H13A	−0.010336	0.678992	0.293384	0.012*	
H13B	−0.068191	0.773773	0.199606	0.012*	
H1WA	0.717 (7)	0.527 (5)	0.037 (4)	0.013 (15)*	
H1WB	0.596 (8)	0.451 (5)	−0.036 (5)	0.039 (17)*	
N1	0.6840 (5)	0.2560 (3)	0.2752 (3)	0.0108 (8)	
N2	0.4008 (5)	0.4211 (3)	0.1594 (3)	0.0084 (7)	
N3	0.2166 (5)	0.7221 (3)	0.2324 (3)	0.0091 (7)	
H3N	0.306 (7)	0.719 (4)	0.293 (4)	0.017 (13)*	
C9	0.3931 (6)	0.5548 (4)	0.1691 (3)	0.0108 (8)	
H9A	0.416736	0.592496	0.106217	0.013*	
H9B	0.497536	0.581169	0.229345	0.013*	
C10	0.1989 (6)	0.5987 (4)	0.1829 (3)	0.0115 (9)	
H10A	0.103663	0.602054	0.114376	0.014*	
H10B	0.150619	0.540629	0.226776	0.014*	
C12	0.4880 (5)	0.8448 (4)	0.1976 (3)	0.0098 (8)	
C11	0.2714 (6)	0.8182 (4)	0.1662 (3)	0.0110 (9)	
H11A	0.199397	0.893211	0.171991	0.013*	
H11B	0.233493	0.791951	0.092846	0.013*	
O2W	0.4169 (6)	0.6478 (4)	0.4259 (3)	0.0250 (9)	
H2WA	0.373 (9)	0.581 (6)	0.441 (5)	0.05 (2)*	
H2WB	0.526 (9)	0.660 (6)	0.449 (5)	0.04 (2)*	
O3W	0.3776 (6)	0.4191 (4)	0.4967 (3)	0.0260 (9)	
H3WA	0.278 (14)	0.409 (10)	0.511 (8)	0.15 (5)*	
H3WB	0.452 (9)	0.390 (6)	0.547 (5)	0.06 (2)*	
O4W	0.8110 (5)	0.6367 (3)	0.5369 (3)	0.0239 (8)	
H4WA	0.865 (8)	0.603 (6)	0.499 (5)	0.04 (2)*	
H4WB	0.806 (9)	0.574 (7)	0.571 (5)	0.06 (2)*	

### Atomic displacement parameters (Å^2^)

**Table:**

	*U*^11^	*U*^22^	*U*^33^	*U*^12^	*U*^13^	*U*^23^
Ga1	0.00710 (17)	0.00922 (19)	0.01104 (19)	0.0001 (2)	0.00244 (14)	−0.0001 (2)
C1	0.014 (2)	0.013 (2)	0.009 (2)	−0.0019 (17)	−0.0004 (17)	0.0032 (16)
O1	0.0069 (14)	0.0131 (16)	0.0119 (15)	−0.0001 (12)	−0.0005 (12)	0.0006 (12)
O2	0.0205 (17)	0.0209 (18)	0.0129 (16)	−0.0102 (14)	−0.0018 (14)	−0.0009 (14)
C2	0.0083 (19)	0.014 (2)	0.014 (2)	0.0032 (17)	0.0043 (18)	0.0001 (18)
O3	0.0074 (14)	0.0141 (16)	0.0213 (17)	0.0017 (13)	0.0038 (13)	0.0035 (13)
C3	0.012 (2)	0.011 (2)	0.018 (2)	0.0029 (17)	−0.0011 (18)	0.0037 (18)
O4	0.0219 (18)	0.023 (2)	0.054 (3)	0.0120 (16)	0.0190 (18)	0.0114 (18)
C4	0.0086 (19)	0.016 (2)	0.024 (3)	−0.0025 (18)	0.0021 (19)	−0.0013 (19)
O5	0.0080 (13)	0.0093 (15)	0.0128 (15)	0.0013 (12)	0.0022 (12)	−0.0041 (12)
C5	0.0103 (19)	0.012 (2)	0.013 (2)	−0.0014 (17)	0.0031 (17)	0.0054 (17)
O6	0.0120 (14)	0.0107 (15)	0.0154 (16)	−0.0012 (12)	0.0007 (13)	−0.0020 (12)
C6	0.0110 (16)	0.0127 (18)	0.0110 (17)	0.001 (2)	0.0044 (14)	0.000 (2)
O7	0.0138 (14)	0.0143 (15)	0.0121 (15)	−0.0054 (13)	0.0039 (13)	0.0009 (12)
C7	0.0068 (16)	0.010 (2)	0.0087 (17)	−0.0003 (16)	0.0006 (14)	−0.0033 (16)
O8	0.0088 (14)	0.0130 (15)	0.0131 (15)	−0.0006 (12)	0.0009 (12)	0.0017 (12)
C8	0.0118 (19)	0.009 (2)	0.009 (2)	−0.0005 (17)	0.0037 (17)	0.0019 (16)
O9	0.0086 (13)	0.0172 (16)	0.0187 (16)	−0.0016 (12)	0.0006 (12)	−0.0037 (13)
O10	0.0080 (13)	0.018 (2)	0.0136 (15)	0.0008 (12)	0.0023 (12)	−0.0027 (12)
O1W	0.0157 (16)	0.0096 (17)	0.0120 (17)	0.0001 (14)	0.0041 (13)	0.0004 (13)
C14	0.0104 (16)	0.0093 (17)	0.0118 (17)	0.004 (2)	0.0025 (14)	0.007 (2)
C13	0.0051 (18)	0.012 (2)	0.013 (2)	0.0008 (17)	0.0033 (16)	−0.0022 (17)
N1	0.0086 (17)	0.0101 (18)	0.0131 (19)	−0.0016 (15)	0.0018 (15)	0.0001 (15)
N2	0.0074 (16)	0.0080 (17)	0.0097 (17)	0.0015 (14)	0.0021 (14)	−0.0005 (14)
N3	0.0052 (16)	0.0086 (17)	0.0129 (19)	−0.0008 (14)	0.0013 (15)	−0.0005 (14)
C9	0.0082 (19)	0.009 (2)	0.016 (2)	−0.0019 (16)	0.0042 (17)	−0.0022 (17)
C10	0.0079 (19)	0.009 (2)	0.018 (2)	−0.0036 (16)	0.0033 (17)	−0.0022 (17)
C12	0.0094 (16)	0.008 (2)	0.0131 (18)	−0.0030 (18)	0.0046 (15)	−0.0025 (18)
C11	0.0099 (19)	0.0107 (19)	0.012 (2)	0.0005 (16)	0.0029 (17)	0.0030 (16)
O2W	0.0194 (19)	0.026 (2)	0.026 (2)	−0.0052 (17)	−0.0019 (17)	0.0104 (17)
O3W	0.0217 (19)	0.033 (2)	0.021 (2)	−0.0020 (17)	0.0021 (17)	0.0110 (17)
O4W	0.0231 (19)	0.020 (2)	0.026 (2)	0.0017 (16)	0.0015 (17)	0.0028 (17)

### Geometric parameters (Å, º)

**Table:**

Ga1—O1W	1.916 (3)	C7—H7A	0.9900
Ga1—O3	1.925 (3)	C7—H7B	0.9900
Ga1—O1	1.933 (3)	O8—C12	1.251 (5)
Ga1—O5	1.964 (3)	O9—C14	1.211 (5)
Ga1—N1	2.081 (4)	O10—C14	1.318 (4)
Ga1—N2	2.156 (3)	O10—H10O	0.87 (6)
C1—N1	1.499 (5)	O1W—H1WA	0.71 (5)
C1—C2	1.517 (6)	O1W—H1WB	0.99 (6)
C1—H1A	0.9900	C14—C13	1.508 (6)
C1—H1B	0.9900	C13—N3	1.493 (5)
O1—C2	1.286 (5)	C13—H13A	0.9900
O2—C2	1.236 (5)	C13—H13B	0.9900
O3—C4	1.305 (5)	N2—C9	1.486 (5)
C3—N1	1.474 (5)	N3—C11	1.497 (5)
C3—C4	1.530 (6)	N3—C10	1.507 (5)
C3—H3A	0.9900	N3—H3N	0.90 (5)
C3—H3B	0.9900	C9—C10	1.526 (5)
O4—C4	1.219 (5)	C9—H9A	0.9900
O5—C8	1.293 (5)	C9—H9B	0.9900
C5—N1	1.486 (5)	C10—H10A	0.9900
C5—C6	1.516 (6)	C10—H10B	0.9900
C5—H5A	0.9900	C12—C11	1.525 (5)
C5—H5B	0.9900	C11—H11A	0.9900
O6—C8	1.236 (5)	C11—H11B	0.9900
C6—N2	1.505 (5)	O2W—H2WA	0.84 (7)
C6—H6A	0.9900	O2W—H2WB	0.77 (6)
C6—H6B	0.9900	O3W—H3WA	0.79 (10)
O7—C12	1.252 (5)	O3W—H3WB	0.81 (7)
C7—N2	1.483 (5)	O4W—H4WA	0.80 (6)
C7—C8	1.517 (6)	O4W—H4WB	0.83 (7)
			
O1W—Ga1—O3	97.21 (13)	O5—C8—C7	117.1 (3)
O1W—Ga1—O1	89.15 (14)	C14—O10—H10O	117 (4)
O3—Ga1—O1	95.91 (12)	Ga1—O1W—H1WA	125 (4)
O1W—Ga1—O5	93.24 (14)	Ga1—O1W—H1WB	118 (3)
O3—Ga1—O5	89.19 (12)	H1WA—O1W—H1WB	111 (5)
O1—Ga1—O5	174.05 (12)	O9—C14—O10	122.6 (4)
O1W—Ga1—N1	174.57 (16)	O9—C14—C13	123.2 (4)
O3—Ga1—N1	83.30 (14)	O10—C14—C13	114.2 (3)
O1—Ga1—N1	85.42 (13)	N3—C13—C14	110.3 (3)
O5—Ga1—N1	92.17 (13)	N3—C13—H13A	109.6
O1W—Ga1—N2	95.26 (13)	C14—C13—H13A	109.6
O3—Ga1—N2	164.97 (13)	N3—C13—H13B	109.6
O1—Ga1—N2	92.62 (12)	C14—C13—H13B	109.6
O5—Ga1—N2	81.75 (12)	H13A—C13—H13B	108.1
N1—Ga1—N2	85.08 (13)	C3—N1—C5	114.1 (3)
N1—C1—C2	113.2 (3)	C3—N1—C1	111.7 (3)
N1—C1—H1A	108.9	C5—N1—C1	113.2 (3)
C2—C1—H1A	108.9	C3—N1—Ga1	104.3 (3)
N1—C1—H1B	108.9	C5—N1—Ga1	106.3 (2)
C2—C1—H1B	108.9	C1—N1—Ga1	106.4 (2)
H1A—C1—H1B	107.7	C7—N2—C9	112.0 (3)
C2—O1—Ga1	116.2 (3)	C7—N2—C6	112.9 (3)
O2—C2—O1	123.3 (4)	C9—N2—C6	109.5 (3)
O2—C2—C1	118.6 (4)	C7—N2—Ga1	104.7 (2)
O1—C2—C1	118.0 (4)	C9—N2—Ga1	112.3 (2)
C4—O3—Ga1	115.8 (3)	C6—N2—Ga1	105.1 (2)
N1—C3—C4	111.1 (4)	C13—N3—C11	112.6 (3)
N1—C3—H3A	109.4	C13—N3—C10	109.4 (3)
C4—C3—H3A	109.4	C11—N3—C10	112.9 (3)
N1—C3—H3B	109.4	C13—N3—H3N	104 (3)
C4—C3—H3B	109.4	C11—N3—H3N	109 (3)
H3A—C3—H3B	108.0	C10—N3—H3N	108 (3)
O4—C4—O3	124.5 (4)	N2—C9—C10	112.5 (3)
O4—C4—C3	119.8 (4)	N2—C9—H9A	109.1
O3—C4—C3	115.7 (4)	C10—C9—H9A	109.1
C8—O5—Ga1	117.3 (3)	N2—C9—H9B	109.1
N1—C5—C6	109.5 (3)	C10—C9—H9B	109.1
N1—C5—H5A	109.8	H9A—C9—H9B	107.8
C6—C5—H5A	109.8	N3—C10—C9	111.5 (3)
N1—C5—H5B	109.8	N3—C10—H10A	109.3
C6—C5—H5B	109.8	C9—C10—H10A	109.3
H5A—C5—H5B	108.2	N3—C10—H10B	109.3
N2—C6—C5	112.8 (3)	C9—C10—H10B	109.3
N2—C6—H6A	109.0	H10A—C10—H10B	108.0
C5—C6—H6A	109.0	O8—C12—O7	126.7 (3)
N2—C6—H6B	109.0	O8—C12—C11	117.3 (4)
C5—C6—H6B	109.0	O7—C12—C11	115.9 (3)
H6A—C6—H6B	107.8	N3—C11—C12	112.1 (3)
N2—C7—C8	111.7 (3)	N3—C11—H11A	109.2
N2—C7—H7A	109.3	C12—C11—H11A	109.2
C8—C7—H7A	109.3	N3—C11—H11B	109.2
N2—C7—H7B	109.3	C12—C11—H11B	109.2
C8—C7—H7B	109.3	H11A—C11—H11B	107.9
H7A—C7—H7B	107.9	H2WA—O2W—H2WB	117 (6)
O6—C8—O5	123.4 (4)	H3WA—O3W—H3WB	101 (8)
O6—C8—C7	119.5 (3)	H4WA—O4W—H4WB	93 (6)
			
Ga1—O1—C2—O2	171.5 (3)	C2—C1—N1—C3	116.1 (4)
Ga1—O1—C2—C1	−8.7 (4)	C2—C1—N1—C5	−113.6 (4)
N1—C1—C2—O2	−176.8 (4)	C2—C1—N1—Ga1	2.9 (4)
N1—C1—C2—O1	3.5 (5)	C8—C7—N2—C9	150.0 (3)
Ga1—O3—C4—O4	172.0 (4)	C8—C7—N2—C6	−85.8 (4)
Ga1—O3—C4—C3	−5.9 (5)	C8—C7—N2—Ga1	28.0 (4)
N1—C3—C4—O4	163.3 (4)	C5—C6—N2—C7	83.8 (4)
N1—C3—C4—O3	−18.7 (5)	C5—C6—N2—C9	−150.7 (3)
N1—C5—C6—N2	51.6 (4)	C5—C6—N2—Ga1	−29.8 (4)
Ga1—O5—C8—O6	178.6 (3)	C14—C13—N3—C11	−70.5 (4)
Ga1—O5—C8—C7	−0.2 (5)	C14—C13—N3—C10	163.1 (3)
N2—C7—C8—O6	160.4 (4)	C7—N2—C9—C10	63.9 (4)
N2—C7—C8—O5	−20.7 (5)	C6—N2—C9—C10	−62.1 (4)
O9—C14—C13—N3	−3.2 (6)	Ga1—N2—C9—C10	−178.5 (3)
O10—C14—C13—N3	179.4 (3)	C13—N3—C10—C9	−169.5 (3)
C4—C3—N1—C5	146.3 (4)	C11—N3—C10—C9	64.3 (4)
C4—C3—N1—C1	−83.8 (4)	N2—C9—C10—N3	158.1 (3)
C4—C3—N1—Ga1	30.7 (4)	C13—N3—C11—C12	138.0 (4)
C6—C5—N1—C3	−158.9 (3)	C10—N3—C11—C12	−97.6 (4)
C6—C5—N1—C1	72.0 (4)	O8—C12—C11—N3	−9.5 (6)
C6—C5—N1—Ga1	−44.5 (4)	O7—C12—C11—N3	171.9 (4)

### Hydrogen-bond geometry (Å, º)

**Table:**

*D*—H···*A*	*D*—H	H···*A*	*D*···*A*	*D*—H···*A*
O10—H10*O*···O8^i^	0.87 (6)	1.67 (6)	2.525 (4)	169 (6)
O1*W*—H1*WA*···O6^ii^	0.71 (5)	1.93 (5)	2.636 (5)	174 (6)
O1*W*—H1*WB*···O7^iii^	0.99 (6)	1.54 (6)	2.524 (5)	174 (5)
O2*W*—H2*WA*···O3*W*	0.84 (7)	1.94 (7)	2.741 (5)	158 (6)
O2*W*—H2*WB*···O4*W*	0.77 (6)	2.09 (6)	2.828 (5)	160 (6)
O3*W*—H3*WA*···O2^i^	0.79 (10)	2.47 (10)	2.934 (5)	119 (9)
O3*W*—H3*WA*···O10^iv^	0.79 (10)	2.37 (10)	3.096 (5)	153 (9)
O3*W*—H3*WB*···O8^v^	0.81 (7)	2.60 (6)	3.215 (5)	134 (5)
O3*W*—H3*WB*···O9^v^	0.81 (7)	2.28 (6)	2.934 (5)	138 (6)
O4*W*—H4*WA*···O2	0.80 (6)	2.00 (6)	2.806 (5)	175 (6)
O4*W*—H4*WB*···O9^v^	0.83 (7)	2.09 (7)	2.911 (5)	168 (7)
N3—H3*N*···O2*W*	0.90 (5)	1.91 (5)	2.737 (5)	152 (4)
C1—H1*A*···O4*W*^vi^	0.99	2.43	3.417 (6)	173
C3—H3*A*···O7^vii^	0.99	2.25	3.197 (5)	159
C3—H3*B*···O10^viii^	0.99	2.52	3.225 (5)	128
C6—H6*B*···O3*W*	0.99	2.53	3.254 (5)	130
C7—H7*B*···O3^i^	0.99	2.28	3.227 (5)	161
C9—H9*B*···O8	0.99	2.53	3.207 (5)	126
C10—H10*A*···O6^ix^	0.99	2.46	3.271 (5)	139
C10—H10*B*···O1^i^	0.99	2.53	3.300 (5)	134
C11—H11*A*···O4^x^	0.99	2.45	3.438 (5)	176
C13—H13*A*···O1^i^	0.99	2.54	3.367 (5)	140
C13—H13*A*···O2^i^	0.99	2.41	3.368 (5)	162
C13—H13*B*···O6^ix^	0.99	2.34	3.150 (5)	139

Symmetry codes: (i) *x*−1, *y*, *z*; (ii) −*x*+1, *y*+1/2, −*z*; (iii) −*x*+1, *y*−1/2, −*z*; (iv) −*x*, *y*−1/2, −*z*+1; (v) −*x*+1, *y*−1/2, −*z*+1; (vi) −*x*+2, *y*−1/2, −*z*+1; (vii) *x*, *y*−1, *z*; (viii) *x*+1, *y*−1, *z*; (ix) −*x*, *y*+1/2, −*z*; (x) *x*−1, *y*+1, *z*.
